# Tryptophan metabolite atlas uncovers organ, age, and sex‐specific variations

**DOI:** 10.1002/2211-5463.70123

**Published:** 2025-09-19

**Authors:** Lizbeth Perez‐Castro, Afshan F. Nawas, Jessica A. Kilgore, Pedro A. S. Nogueira, M.Carmen Lafita‐Navarro, Paul H. Acosta, Roy Garcia, Noelle S. Williams, Maralice Conacci‐Sorrell

**Affiliations:** ^1^ Department of Cell Biology University of Texas Southwestern Medical Center Dallas USA; ^2^ Department of Biochemistry University of Texas Southwestern Medical Center Dallas USA; ^3^ Lyda Hill Department of Bioinformatics University of Texas Southwestern Medical Center Dallas USA; ^4^ Hamon Center for Regenerative Science and Medicine University of Texas Southwestern Medical Center Dallas USA; ^5^ Harold C. Simmons Comprehensive Cancer Center University of Texas Southwestern Medical Center Dallas USA

**Keywords:** atlas, indole‐3‐pyruvate, kynurenine, metabolism, serotonin, tryptophan

## Abstract

Tryptophan (Trp) is the largest and most structurally complex amino acid, yet it is the least abundant in the proteome. Its distinct indole ring and high carbon content allow it to give rise to several biologically active metabolites, including serotonin, kynurenine (Kyn), and indole‐3‐pyruvate (I3P). Dysregulation of Trp metabolism has been implicated in a range of diseases, from depression to cancer. Investigating Trp and its metabolites in healthy tissues provides insight into how disease‐associated disruptions may be targeted selectively while preserving essential physiological functions. Whereas previous studies have typically focused on individual organs or single metabolic branches, our analysis spans 12 peripheral organs, the central nervous system, and serum in male and female (C57BL/6) mice across three life stages: young (3 weeks), adult (54 weeks), and aged (74 weeks). We identified striking tissue‐, sex‐, and age‐specific differences in Trp metabolism, including elevated levels of I3P and Kyn, both linked to tumor growth, in aging males. We also compared Trp metabolite profiles in tissues from mice fed a control defined diet versus a Trp‐deficient diet for three weeks. This intervention led to a marked reduction in circulating Trp and its metabolites, with more modest effects observed in the liver and central nervous system. These findings underscore the importance of organ‐specific and diet‐sensitive analyses of Trp metabolism for understanding its role in both normal physiology and disease. Establishing baseline levels of Trp metabolites across tissues may also provide a foundation for identifying organ‐specific metabolic reprogramming in cancer and other illnesses.

## Graphical abstract

Tryptophan metabolites were analyzed across twelve organs, the central nervous system, and serum in male and female mice at three life stages. We found tissue‐, sex‐, and age‐specific differences, including increased indole‐3‐pyruvate and kynurenine in aging males. A tryptophan‐deficient diet reduced circulating metabolites, highlighting organ‐ and diet‐sensitive regulation of tryptophan pathways in health and disease.

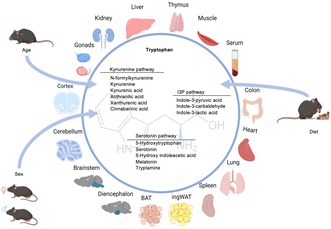

**Abbreviations**
3HAA: 3‐hydroxyanthranilic acid
5‐HIAA: 5‐hydroxyindole acetic acid
5‐HTP: L‐5‐hydroxytryptophan
5‐hydroxytryptamine, 5‐HT: serotonin
AA: amino acid diet
AA: anthranilic acid
AFMID: arylformamidase
AHR: aryl hydrocarbon receptor
BAT: brown adipose tissue
CA: cinnabarinic acid
DDC: dopa decarboxylase
I3A: indole‐3‐carboxaldehyde
I3P: Indole‐3‐pyruvate
IDO1: indoleamine 2,3‐dioxygenase 1
IDO2: indoleamine 2,3‐dioxygenase 2
IL4I1: interleukin 4‐induced 1
ILA: indole‐3‐lactic acid
ingWAT: inguinal white adipose tissue
IS: internal standard
KA: kynurenic acid
KAT: kynurenine aminotransferase
Kyn: kynurenine
KYNU: kynureninase
MAO: monoamine oxidase
MAOA: monoamine oxidase A
NAD+: nicotinamide adenine dinucleotide
NFAA: N‐formylanthranilic acid
NFK: N‐formylkynurenine
PBS: phosphate‐buffered saline
PCA: principal component analysis
QPRT: quinolinate phosphoribosyltransferase
TDO2: tryptophan 2,3‐dioxygenase
TF: Trp‐free diet
TPH1, TPH2: tryptophan hydroxylases
Trp: tryptophan
XA: xanthurenic acid

Tryptophan (Trp), one of the nine essential amino acids, is distinguished by its large size and unique chemical structure, featuring the highest carbon count among essential amino acids and an indole ring. This ring grants Trp hydrophobic properties that are critical in protein structure and protein interactions [1, 2, 3]. Trp is the least abundant amino acid in the proteome representing only an average of 1.3% of the protein content [1]. Therefore, the majority of Trp molecules serve as precursors for a wide range of downstream catabolites [4] that can carry specific biological activities including immunoregulation and neuronal signaling [2, 4]. Thus, it is not surprising that many of these catabolites are implicated in diseases such as cancers, neurological disorders, and digestive disorders [2, 5, 6, 7, 8, 9, 10, 11, 12].

Trp can be metabolized through three primary pathways: the serotonin pathway, which is predominantly active in the central and peripheral nervous systems; the kynurenine (Kyn) pathway, mainly functioning in the liver; and the indole‐3‐pyruvate (I3P) pathway, whose function is not entirely understood but was recently shown to promote tumor growth by repressing immune cells [13, 14]. The most extensively studied Trp‐metabolizing pathway is the Kyn pathway, which generates a range of biologically active metabolites, including Kyn, kynurenic acid (KA), cinnabarinic acid (CA), xanthurenic acid (XA), and nicotinamide adenine dinucleotide (NAD+) [15, 16, 17, 18]. Imbalance of these metabolites is linked to several diseases like dementia, schizophrenia, and cancer (Table 1). The levels and activity of specific enzymes within the Kyn pathway determine both the production rate and stability of Trp metabolites. While the I3P pathway is still understudied, it has been found to play a role in glioblastoma and liver cancer [14, 19].

**Table 1:** Description of Trp and its metabolites: abbreviations, functions, related enzymes, and disease associations.

Metabolite	Abbreviation	Function	Producing enzyme	Disease association	References
Tryptophan	Trp, W	Protein synthesis Metabolite production	N/A		
Kynurenine	Kyn	AHR ligand Immune cell inhibition Involved in inflammatory response	TDO2, IDO1, IDO2, AFMID	Cancer, colitis	[2, 21]
Kynurenic acid	KA	Allosteric inhibitor of α7 nicotinic receptors Glutamate receptor antagonist Inhibits dopamine release in the striatum Anti‐inflammatory	KAT	Schizophrenia	[21, 22]
Anthranilic acid	AA	Neurotoxicity (uptake triggers apoptosis) Inhibits cytotoxic activity of macrophages	KYNU	Depression, Dementia	[10, 23, 24]
Xanthurenic acid	XA	Activates metabotropic glutamate receptors mGlu2/3 Stimulates dopamine release	KAT	Schizophrenia	[12, 25]
Cinnabarinic acid	CA	Agonist of type 4 metabotropic glutamate (mGlu4) receptors Neuroprotective Hepatoprotective and anti‐steatosis	Non‐enzymatic Possibly by other enzymes in the pathway	Unknown	[25, 26]
5‐Hydroxy Tryptophan	5‐HTP	Anti‐inflammatory ROS scavenger	TPH1/2		[27]
Serotonin		Neurotransmitter Modulate gastrointestinal motility	DDC	Depression	[2, 7]
5‐Hydroxy indole acetic acid	5‐HIAA	Used to estimate the levels of serotonin in humans to determine signs of depression	MAOA	Carcinoid tumors, Autism spectrum disorder, Celiac disease	[28, 29, 30]
Indole‐3‐pyruvic acid	I3PA	AHR ligand Immune cell inhibition	IL4I1	Cancer	[14]
Indole‐3‐ carboxaldehyde	I3A	AHR ligand Ameliorates intestinal barrier damage	Unknown in humans	Unknown	[16]
Indole‐3‐lactic acid	ILA	AHR ligand Anti‐inflammatory in immature enterocytes	Unknown in humans Aromatic lactate dehydrogenase (bacteria)	Unknown	[13]
Nicotinamide adenine dinucleotide	NAD+	Energy metabolism Cellular signaling DNA repair	QPRT	Metabolic disorders, Age‐related diseases, Neurological disorders	[18, 31, 32]

The initial step of the Kyn pathway can be catalyzed by indoleamine 2,3‐dioxygenase 1 (IDO1), indoleamine 2,3‐dioxygenase 2 (IDO2), and tryptophan 2,3‐dioxygenase (TDO2) [33, 34]. Previous studies have found Kyn and one or more of these Kyn‐generating enzymes upregulated in tumors of several organs [35, 36, 37, 38, 39, 40]. Kyn serves as a ligand for the transcription factor, aryl hydrocarbon receptor (AHR) [41, 42], which promotes growth pathways in cancer cells [11, 19, 35, 43]. The newly identified metabolite I3P is generated via the activity of the enzyme interleukin 4‐induced 1 (IL4I1), a secreted L‐amino acid oxidase [14]. I3P's downstream metabolites are also proposed to function as ligands for AHR [14, 19]. Kyn, and to a lesser extent some of its metabolites such as KA, can promote immune evasion by suppressing T cell proliferation and activation, thereby creating a more permissive environment for disease progression, including tumor growth. [14, 20, 44]. NAD, the final product of the Kyn pathway, plays a central role in cellular homeostasis by cycling between its reduced (NADH) and oxidized (NAD^+^) forms. This redox cycling enables NAD to facilitate numerous enzymatic reactions, supporting essential processes such as energy production, metabolism, and cellular signaling [16, 31, 32].

Tryptophan hydroxylases (TPH1, TPH2) are essential for serotonin production, with most synthesis occurring in the peripheral nervous system specifically in the distal gastrointestinal tract (90%) and a smaller amount in the central nervous system (10%) [45]. TPH initiates the rate‐limiting step that converts Trp into serotonin. TPH1 is expressed in enterochromaffin cells in the gut, while TPH2 is present in serotonergic neurons of the central and enteric nervous systems. Both TPH1 and TPH2 catalyze the transformation of Trp into L‐5‐hydroxytryptophan (5‐HTP), which is then converted into serotonin (5‐hydroxytryptamine, 5‐HT) by L‐amino acid decarboxylase [45]. In the pineal gland, TPH1 also converts Trp into serotonin, which can subsequently be converted into melatonin. Furthermore, serotonin can be catabolized by monoamine oxidase (MAO) into 5‐hydroxyindole acetaldehyde, and further processed by aldehyde dehydrogenase into 5‐hydroxyindole acetic acid (5‐HIAA), which is excreted in urine [45]. The complexity of Trp metabolism is further compounded by the gut microbiome, which directly and indirectly influences Trp catabolite production, leading to associated changes in behavior and cognition. Consequently, the gut microbiome has attracted significant interest as a therapeutic target for neurological and psychiatric disorders, where Trp and its metabolites are central players [45] (Table 1).

Our previous work identified an upregulation of enzymes involved in Kyn production and, thus, an increase in Kyn levels in colon cancer [11, 43], which led to the activation of AHR [8, 11, 43]. In contrast, MYC‐driven liver tumors exhibit repression of these Kyn pathway enzymes, along with lower levels of Kyn [19]. Interestingly, liver tumors upregulate IL4I1 and its product I3P, which acts as a potent oncometabolite in the liver [19]. We then surmised that to understand the disease‐specific alterations in Trp and its metabolites, we first needed to define their physiological production and function in normal tissues. To better characterize Trp utilization in healthy tissues, we employed LC‐MS/MS to quantify 17 Trp metabolites across the three main Trp‐metabolizing pathways. We measured these metabolites in circulation and across visceral organs and the central nervous system in both male and female C57BL/6 mice to compare sex‐ and age‐related differences in metabolite levels. Although we aimed to measure 17 metabolites, only 13 yielded detectable peaks. To our knowledge, this represents the first comprehensive *in vivo* quantification of Trp metabolites across multiple tissues. This platform provides a valuable reference dataset for researchers investigating Trp metabolism in both physiological and pathological contexts.

## Methods

### Mice

Mice were housed together in a barrier facility at 22°C and 30–70% humidity. Colon, heart, lung, spleen, kidney, liver, gonads, brown adipose tissue (BAT), muscle (gastrocnemius), thymus, inguinal white adipose tissue (ingWAT), brain, and blood were collected from 3‐, 53‐, and 74‐week‐old mice. Male and female C57BL/6 mice were utilized (The Jackson Laboratory Strain#:000664). Mouse age groups were selected according to Jackson Laboratory's age equivalency guidelines (https://resources.jax.org/white‐papers/whitepaper‐aged‐b6), aiming to represent the critical human life stages of adolescence, peak adulthood, and early elderly within our study. All procedures used in this study are approved by the Institutional Animal Care and Use Committee of the University of Texas Southwestern Medical Center under the protocol APN 2017‐101798.

### Metabolite extraction

Organs were collected from the all‐mouse groups within a two‐hour window to minimize variability. The first group was processed around 10 a.m., and the second group around 1 p.m. Tissues were immediately flash‐frozen in liquid nitrogen and stored at –80°C until LC‐MS/MS analysis. Blood samples were incubated at room temperature for 30 min, then centrifuged at 3500 ***g*** for 10 min to isolate the serum. To extract metabolites from the different diets, equal weights of food pellets were ground into a fine powder using a mortar and pestle, then incubated overnight in 80% methanol with end‐over‐end mixing. The resulting mixture was filtered through a 22‐micron filter before analysis.

### Trp metabolite quantification

LC‐MS/MS evaluation of Trp metabolite concentrations was performed by the UTSW Preclinical Pharmacology Core as previously described [46] with the following minor modifications. Mouse tissues were homogenized in PBS prior to extraction with 80% final volume methanol. Serum was extracted similarly. Tissue concentrations were normalized to wet tissue weight. Tissues were homogenized in a 3‐fold volume of phosphate‐buffered saline (PBS) (3 × weight of tissue in g = vol PBS in mL; total homogenate volume (in mL) = 4 × weight of tissue) using BeadBug microtube homogenizer prefilled tubes with 3.0 mm Zirconium beads (Sigma Cat #Z763802), run for 2 min at 2800 ***g***. Then, 50 μL of each tissue homogenate or serum sample were mixed with 200 μL methanol, vortexed for 15 seconds, incubated at RT for 10 min and spun in a tabletop, chilled centrifuge for 5 min at 16 100 × ***g***. Supernatants were transferred to Eppendorf tubes and dried down using a SpeedVac under no heat. The dried samples were resuspended in 0.1 mL ddH_2_O + 25 ng·mL^−1^ tolbutamide internal standard (IS) + 10 ng·mL^−1^ d5 Trp IS. Standards were made by combining equal amounts of tissue lysates (in final resuspension solution) as a background matrix. The standard mix was diluted with resuspension solution 1 : 1000 for Kyn, KA, CA, 3HAA, 5HTP, and XA standards; 1 : 5000 for Trp, N‐formylkynurenin (NFK), melatonin, 5HIAA, and serotonin standards; 1 : 5000 for AA and I3CA standards; and 1 : 5000 for NFAA, I3LA, I3PA, and tryptamine standards of diluted standard mix (100 μL) was spiked with 1 μL of the appropriate standard at varying concentrations. Samples were transferred to a low volume 96‐well HPLC plate and analyzed by LC‐MS/MS. Tissues and serum were processed separately and run in two batches. Dilutions and reruns were conducted as needed.

### Principal component analysis (PCA) plots

PCA was performed using the MetaboAnalyst platform (https://www.metaboanalyst.ca), a widely used web‐based tool for metabolomics data processing and statistical analysis. The metabolomics data was input as a CSV file and the data was log‐transformed using the website to stabilize variance and approximate normality, followed by auto‐scaling (mean‐centering and division by the standard deviation) to ensure comparability across features. Then, PCA was done under the one factor statistical analysis for each brain area, and the plot was displayed using the 2D setting.

### Quantification and statistical analyses

Heatmaps were generated using GraphPad and Python Software Foundation (Python Programming Language) after normalizing the data by the max, applying the formula *X*_norm_ = (*X*)/(*X*_max_) for each metabolite this will give a range between 0 to 1. Two‐way ANOVA with Tukey's multiple tests comparison was performed, comparing the mean of each cell to every other cell (*P < 0.05). Finally, significant and near‐significant *q*‐values from sex differences were determined by multiple *t*‐tests across the different age groups. Additionally for accurate comparison density of mouse serum was estimated to be approximately 1.025 g·mL^−1^, which is consistent with reported values for mammalian serum. Using this density, all measurements from ng·mL^−1^ (per volume) were converted to ng·g^−1^ (per mass) to allow for more accurate comparisons across samples (Figures 2, S2, S3).

## Results

### Comprehensive mapping of Trp metabolites across ages, sexes, and tissues

**Fig. 1:**
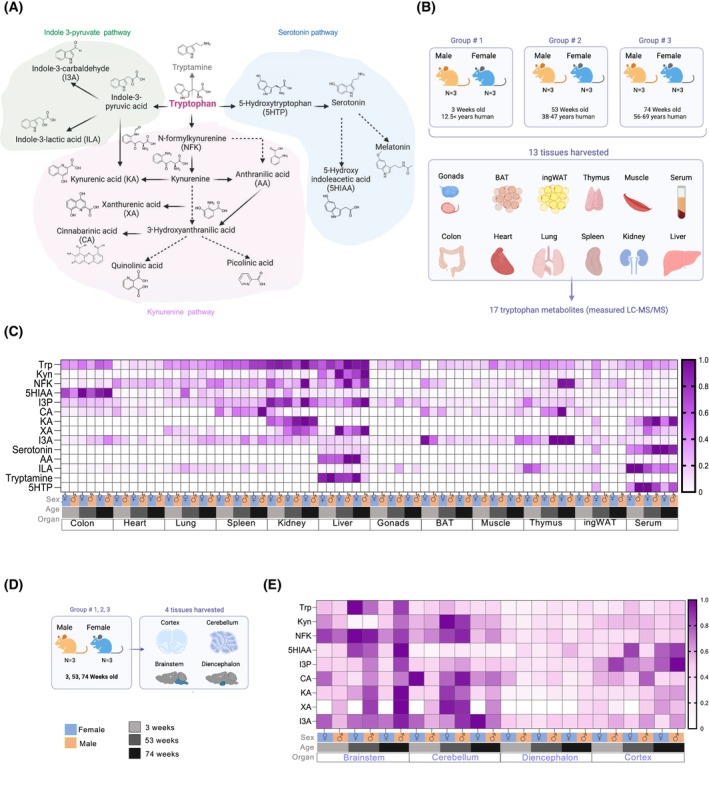
Comprehensive mapping of Trp metabolites across ages, sexes, and tissues. (A) Summary of Trp metabolism pathway and the Trp metabolites quantified by LC‐MS/MS. (B) Schematic of experiment. Tissues were harvested from 3‐, 53‐, and 74‐week‐old male and female C57BL/6 mice. Tissues were flash‐frozen for later processing through LC‐MS/MS. (C) Heatmap of all metabolites in the tissues after being normalize by metabolite (min‐max normalization). (D) Schematic of experiment for the collection of brain areas. Tissues were harvested from 3‐, 53‐, and 74‐week‐old male and female C57BL/6 mice and then processed through LC‐MS/MS. (E) Heatmap of all metabolites in the brain areas.

With the goal of generating an atlas of Trp metabolites *in vivo*, we employed LC‐MS/MS (Fig. S1A–D) to precisely quantify 17 Trp catabolites across the three main Trp‐metabolizing pathways (Fig. 1A). Although we aimed to measure 17 metabolites, only 13 had detectable peaks. We quantified the metabolites in the Kyn pathway: Kyn, N‐formylkynurenine (NFK), KA, anthranilic acid (AA), N‐formylanthranilic acid (NFAA), quinolinic acid, picolinic acid, 3‐hydroxyanthranilic acid (3HAA), and CA; in the serotonin pathway: serotonin, melatonin, 5‐HIAA, and 5‐HTP; and in the I3P pathway: I3P, indole‐3‐carboxaldehyde (I3A), and indole‐3‐lactic acid (ILA) (Fig. 1A). Additionally, we quantified the microbiome‐derived metabolite tryptamine (Fig. 1A). Table 1 summarizes the known functions and regulators of these metabolites.

Using freshly excised, snap‐frozen mouse tissues (Fig. 1B), we measured Trp and its metabolites across a comprehensive set of organs, including gonads, liver, spleen, muscle, colon, heart, lung, kidney, brown adipose tissue (BAT), thymus, inguinal white adipose tissue (ingWAT), and serum from male and female mice at three distinct ages (life stages): 3 weeks (approximating human preadolescence at 12.5 years), 53 weeks (representing human adult age, 38‐47 years), and 74 weeks (representing human ages 56–69 years) (Fig. 1B,C). Additionally, we examined the same Trp metabolites in segmented regions of the central nervous system: cortex, cerebellum, diencephalon, and brainstem of the same mice (Fig. 1D).

Although Trp, I3P, and Kyn were present in most tissues, certain metabolites were either absent or undetectable in mouse tissues resulting in only 13 detectable metabolites. Melatonin was absent in C57BL/6 mice, 3HAA and 5HTP were only detectable in serum, and NFAA was undetectable in all tissues (Fig. 1A–E). These metabolites were not included in further analyses. Max normalization of each metabolite was performed to generate a heatmap displaying the relative levels of each Trp metabolite in both sexes across all organs at the time points sampled (Fig. 1C,E). The heatmaps revealed tissue‐specific patterns in Trp metabolite composition, with nearly all metabolites detected in the serum, indicating their potential to circulate within organs.

Trp levels were highest in the liver, kidney, and spleen, while metabolites in the Kyn pathway were most abundant in the liver (Fig. 1C). I3P was highest in the liver and kidney (Fig. 1C). The liver also displayed the largest amounts of tryptamine and anthranilic acid (Fig. 1C). Circulating Trp levels were markedly lower than those in the liver, kidney, lung, and colon, suggesting rapid uptake by these organs (Fig. 1C). Serotonin and its precursor 5‐HTP levels were highest in circulation, indicating a broad, whole body signaling role for serotonin pathway metabolites. Additionally, the level of the serotonin breakdown product 5‐HIAA was highest in the colon, where serotonin‐producing enterochromaffin cells are located (Fig. 1C). Melatonin, 3HAA, 5HTP, and NFAA were undetectable in these tissues, and therefore, they were not further analyzed. In the central nervous system (cortex, cerebellum, brainstem, and diencephalon; Fig. 1D), 9 out of the 17 metabolites were detected. Among these, Trp, I3P, Kyn, the Kyn precursor NFK, and the serotonin product 5‐HIAA were the most abundant (Fig. 1E). The heatmap revealed a clear spatial specification pattern for these metabolites, with the diencephalon showing the lowest levels of Trp and its related metabolites (Fig. 1E). For all figures, all metabolites measurable in the 3 replicates are for each data set.

### Organ‐ and tissue‐specific variation in Trp metabolite abundance

**Fig. 2:**
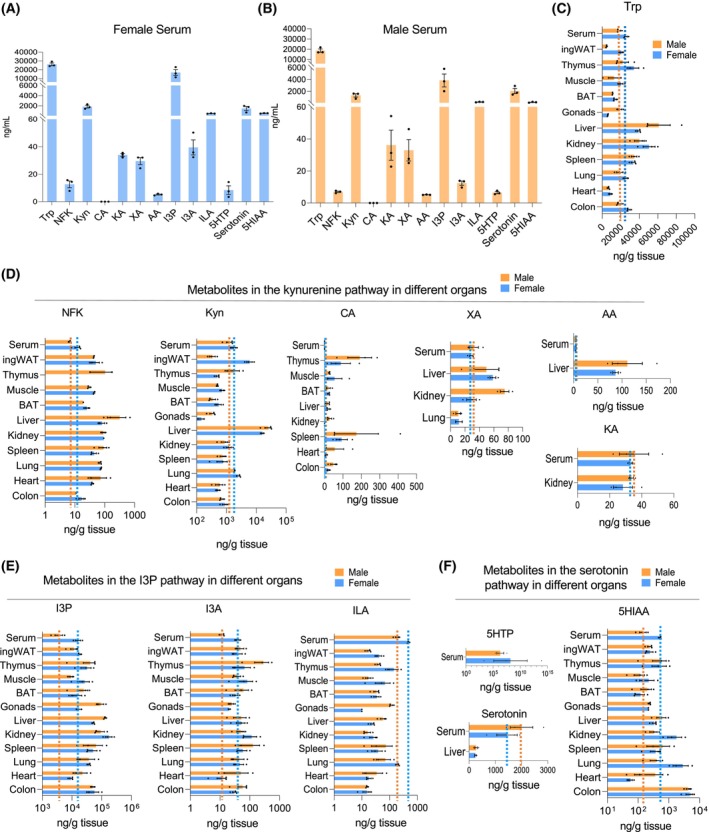
Abundance of Trp metabolites across different organs in adult tissues compared to serum. (A) Abundance (ng·mL^−1^) of all Trp‐derived metabolites by LC‐MS/MS across different tissues in the serum of females. (B) Abundance (ng·mL^−1^) of all Trp‐derived metabolites by LC‐MS/MS across different tissues in the serum of males. (C) Abundance (ng·g^−1^ of tissue) of Trp metabolites across all tissues compared to the amount circulating in serum (ng·g^−1^) (dotted line). (D) Abundance (ng·g^−1^ of tissue) of Kyn pathway metabolites across all tissues compared to the amount circulating in serum (ng·g^−1^) (dotted line). (E) Abundance (ng·g^−1^ of tissue) of I3P pathway metabolites across all tissues compared to the amount circulating in serum (ng·g^−1^) (dotted line). (F) Abundance (ng·g^−1^ of tissue) of serotonin pathway metabolites across all tissues compared to the amount circulating in serum (ng·g^−1^) (dotted line). The density of mouse serum was estimated to be approximately 1.025 g·mL^−1^, which is consistent with reported values for mammalian serum. Using this density, all measurements from ng·mL^−1^ (per volume) were converted to ng·g^−1^ (per mass) to allow for more accurate comparisons across samples. (A–F) Errors bars indicate the mean with SEM; Females *N*=3, Males *N*=3.

Quantitative comparison of Trp metabolites across various organs and circulation in 53‐week‐old (adult) male and female mice was performed to map the differential distribution and establish a reference landscape of baseline Trp metabolism in adult. We compared the levels of all metabolites in circulation (serum) to identify the organs with higher Trp uptake or decreased processing (Fig. 2A,B). Trp levels were noticeably higher in the liver, kidney, and spleen, suggesting that these organs have a significant need for Trp (Fig. 2C). Conversely, ingWAT and the heart exhibited Trp levels that were lower than serum levels (Fig. 2C). In addition to higher levels of Trp, the liver and kidneys also predominantly featured elevated levels of metabolites of the Kyn pathway, except for CA, which was most abundant in the spleen and thymus (Fig. 2D). CA concentrations were higher in organs than serum. NFK was also higher in all male organs than serum, but this trend was reversed in females (Fig. 2D). Although AA and KA were present in the serum, these metabolites were only detectable in the liver and kidneys, respectively (Fig. 2D). Kyn levels were particularly elevated in the liver. XA and AA were more abundant in the liver than in the bloodstream.

Among the I3P pathway metabolites, I3P and I3A were generally higher than serum levels across most organs, whereas ILA did not exceed circulating levels (Fig. 2E). The levels of most metabolites in the serotonin pathway were lower than in the organs than serum, except 5HIAA levels were higher in the serum than in the colon, indicating localized metabolic activity (Fig. 2F). The most prevalent metabolites across all examined peripheral organs in both females and males included Trp, Kyn, I3P, and 5‐HIAA, underscoring their pivotal roles in systemic and organ‐specific function (Figs 1, 2). These data suggest that metabolites with higher levels in specific organs than serum may be more efficient at production and suggest functional specializations within those organs. Some of these trends are visible at 3‐week and 74‐week‐old mice (Figs S2, S3). On the other hand, certain metabolites including CA, XA, and AA decreased as the mice aged (Fig. S3D). Overall, the liver and kidney had the highest expression of Trp‐derived metabolites (Figs 2, S2, S3).

### Sex‐specific variation in Trp metabolite levels

**Fig. 3:**
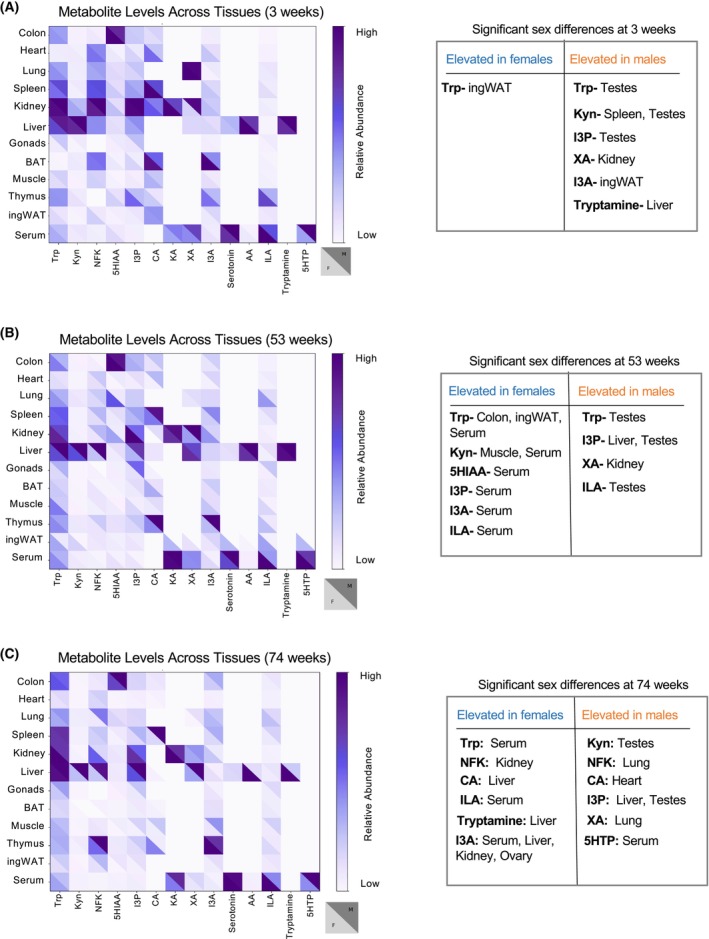
Sex variation of Trp metabolite levels. (A) Heatmap highlighting male and female differences of Trp metabolite abundance across different tissues in 3‐week‐old mice. (B) Heatmap highlighting male and female differences of Trp metabolite abundance across different tissues in 53‐week‐old mice. (C) Heatmap highlighting male and female differences of Trp metabolite abundance across different tissues in 74‐week‐old mice.

To uncover sex specificities, we compared the levels of all Trp metabolites between the sexes at each age stage (Fig. 3). Male and female mice at 3 weeks of age had similar levels of Trp metabolites across tissues (Fig. 3A); however, some organs in older mice exhibited differing metabolite levels between sexes (Fig. 3B,C), suggesting that aging affects Trp metabolism in a sex‐dependent manner. Young male mice exhibited higher levels of Trp metabolites than female mice of the same age: Kyn in the spleen, tryptamine in the liver, I3A in ingWAT, and KA in the kidney (Fig. 3A). In contrast, female mice had higher levels of Trp in the ingWAT (Fig. 3A). Compared with adult male serum, adult female serum had significantly higher levels of Trp metabolites (Fig. 3B). Notably, males had higher levels of I3P in the liver and gonads, I3A in the ingWAT and XA in the kidney than females (Fig. 3B). In aged mice, females exhibited markedly higher levels of I3A in the liver, kidney, serum, and gonads, along with other metabolites like CA and NFK when compared to aged males. Conversely, aged male mice maintained significantly higher levels of I3P in the liver and gonads (Fig. 3C).

### Alterations in Trp metabolite levels during aging

To identify sex‐ and organ‐specific Trp‐metabolic trends across age, we generated PCA plots (Fig. S4). The liver plot revealed a distinct pattern of Trp metabolites in older male mice while BAT and colon plots displayed more modest age differences (Fig. S4). At the metabolite level, concentrations of Kyn and I3P increased with age in the livers of aged males, while ILA concentrations were higher in adult male mice compared to young males (Table 2, Fig. S5A). Trp, Kyn, I3P, and I3A concentrations in the colon were higher in older male mice than in young or adult mice (Fig. S5B). Trp and Kyn concentrations in the colon of female mice also displayed significant changes: Trp concentration increased with age while Kyn showed a significant change only between young and adult mice (Table 2, Fig. S5B). Both I3P and I3A levels in the heart significantly decreased in female mice as they reach adulthood, meanwhile Trp and Kyn levels increase with age (Table 2, Fig. S5C). A clear metabolic switch in BAT occurred between young and adult mice; Trp dramatically increased in adulthood while Trp catabolites I3A, NFK, Kyn, and CA significantly decreased (Table 2, Fig. S5D), suggesting that Trp metabolism may play a role in BAT growth in young mice. Further studies are necessary to determine the specific function of these metabolites in BAT.

**Table 2:** Changes in Trp metabolite pathways with aging, comparing 3‐, 54‐, and 72‐week‐old mice.

Organs	Tryptophan	Kynurenine pathway	Indole‐3‐pyruvic acid pathway
Liver Female			+
Liver Male		+++	+++
Colon Female	+	++	+
Colon Male	+++	+	+++
Heart Female	++	++	–
Heart Male	++	++	−
BAT Female	++	–	−
BAT Male	++	–	−

A plus sign (+) indicates that one or more metabolites within the pathway were higher than the previous age group (multiple + represent a larger, significant difference); a minus sign (−) indicates that one or more metabolites were less than the previous age group (multiple—represent a larger significant difference). The proportion of the difference is indicated by the number of plus or minus signs, with the largest differences represented by multiple symbols. Also see Fig. S5.

### Age‐ and sex‐dependent variations in brain Trp metabolite

**Fig. 4:**
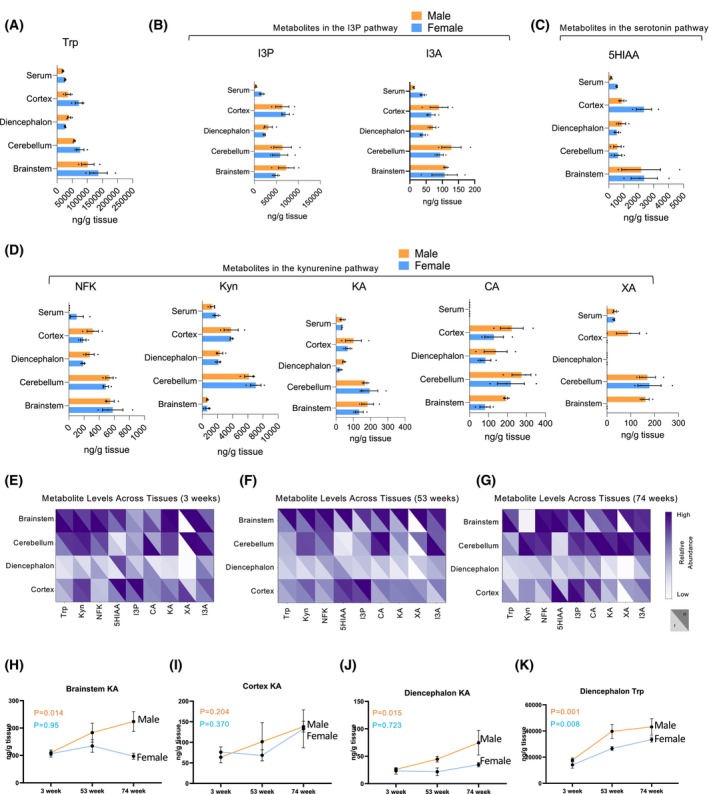
Trp metabolite levels in the brain across different ages and sexes. (A) Amounts (ng·g^−1^) measured by LC‐MS/MS of Trp across different brain regions. (B) Abundance (ng·g^−1^) of metabolites in the I3P pathway by LC‐MS/MS across different brain regions. (C) Abundance (ng·g^−1^) of metabolites in the serotonin pathway by LC‐MS/MS across different brain regions. (D) Abundance (ng·g^−1^) of metabolites in the Kyn pathway by LC‐MS/MS across different brain regions. (E) Heatmap highlighting male and female differences of Trp metabolite abundance across different brain regions in 3‐week‐old mice. (F) Heatmap highlighting male and female differences of Trp metabolite abundance across different brain regions in 53‐week‐old mice. (G) Heatmap highlighting male and female differences of Trp metabolite abundance across different brain regions in 74‐week‐old mice. (H) Significant changes in abundance for KA across aging in the brainstem. (I) Significant changes in abundance for KA across aging in the cortex. (J) Significant changes in abundance for KA across aging in the diencephalon. (K) Significant changes in abundance for Trp across aging in the diencephalon. (A–D) Errors bars indicate the mean with SEM; females *N*=3, males *N*=3. (H–K) Errors bars indicate the mean with SEM; *P* value was done using multiple comparison two‐way ANOVA Age 3 weeks (*N*=6) Females *N*=3, Males *N*=3; Age 53 weeks (*N*=6) Females *N*=3, Males *N*=3; Age 74 weeks (*N*=6) Females *N*=3, Males *N*=3.

By comparing the levels of Trp metabolites in the various regions of the brain with those circulating in the serum at age 53 weeks (Fig. 4A–D), we found that Trp concentrations were notably higher in the cortex, cerebellum, and brainstem than in circulation (Fig. 4A). In males, all metabolites, except for Kyn in the brainstem and XA in the diencephalon and other brain regions, exhibited higher concentrations in the central nervous system than in the serum (Fig. 4A–D). To identify sex specificities, we compared all Trp metabolites between sexes at each age stage (Fig. 4E–G). XA displayed clear sex differences in various brain regions at all 3 ages (Fig. 4E–G). At 3 weeks of age, Trp was higher in the brainstem of females than males (Fig. 4E). At 53 weeks, males had higher concentrations of Trp (Fig. 4F). I3P was higher in the brainstem of males than in females at 74 weeks (Fig. 4G). Additionally, PCA plots did not reveal age‐related trends in sex specificities of metabolites except for the brainstem that had some shifts between males and females (Fig. S6). Overall, the brainstem and cerebellum had the highest levels of Trp‐derived metabolites. Additionally, we examined aging‐related trends and found no notable changes across the brain regions, except for KA, which increased with age in both males and females (Fig. 4H–K).

### Dietary intake of Trp and its metabolites may impact their concentrations in tissues

**Fig. 5:**
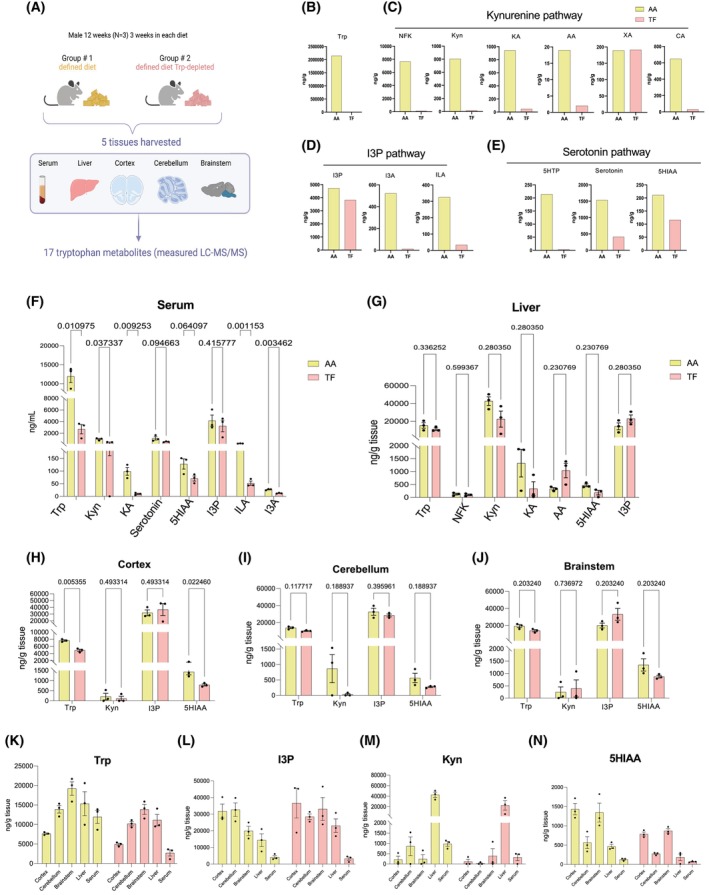
Quantification of Trp metabolites in adult mice. (A) Schematic of the experimental design. Nine‐week‐old male C57BL/6 mice were fed one of three diets for 3 weeks: standard chow, an amino acid‐defined (AA) diet, or a Trp‐depleted (TF) diet. At the end of the study, serum, liver, cortex, cerebellum, and brainstem were collected for metabolomic analysis by LC‐MS/MS. (B) Quantification of Trp levels across the different diets using LC‐MS/MS. (C) Quantification of Kyn pathway metabolites across the different diets using LC‐MS/MS. (D) Quantification of I3P pathway metabolites across the different diets using LC‐MS/MS. (E) Quantification of serotonin pathway metabolites across the different diets using LC‐MS/MS. (F) Tissue‐specific analysis of Trp‐derived metabolites in the serum of mice AA or TF diets. (G) Tissue‐specific analysis of Trp‐derived metabolites in the liver of mice fed AA or TF diets. (H) Tissue‐specific analysis of Trp‐derived metabolites in the cortex of mice fed AA or TF diets. (I) Tissue‐specific analysis of Trp‐derived metabolites in the cerebellum of mice fed AA or TF diets. (J) Tissue‐specific analysis of Trp‐derived metabolites in the brainstem of mice fed AA or TF diets. (K) Trp absolute levels across the different organs. (L) I3P absolute levels across the different organs. (M) Kyn absolute levels across the different organs. (N) 5HIAA absolute levels across the different organs. (F–J) Statistical analysis was performed using multiple t‐tests comparing AA and TF groups. *P < 0.05.

To determine how diet influences tryptophan metabolism, the levels of 17 tryptophan metabolites were quantified in mice maintained on two diets: (1) a defined amino acid diet containing all amino acids and (2) an otherwise identical diet lacking Trp (Fig. 5A, Table S1), both previously used to modulate tryptophan availability in a mouse model of liver cancer [19]. Relative metabolite levels for each diet are shown in Fig. 5B–E. After three weeks on either the complete or Trp‐free diet, circulating levels of all metabolites were reduced, except for serotonin and I3P (Fig. 5F). Most metabolites appeared to trend toward downregulation under Trp restriction in the liver, but only KA reached statistical significance. This suggests that hepatic Trp metabolites may be relatively stable in normal liver, and that a longer duration of dietary depletion may be required to produce more substantial changes. In contrast, nearly all metabolites, except for I3P, in various brain regions appeared to trend toward a downregulation in mice fed the Trp‐free diet (Fig. 5H–J). Overall, Trp‐derived metabolites were lower in the serum, liver, and brain tissues upon dietary Trp restriction (Fig. 5K–N).

In summary, our study presents the first comprehensive atlas of Trp metabolism across multiple organs, ages, and sexes in mice, revealing profound tissue‐specific, age‐related, and sex‐dependent differences in Trp‐derived metabolites. Using targeted LC‐MS/MS analysis, 17 Trp catabolites, including Kyn, I3P, and serotonin pathway metabolites were profiled in 12 peripheral organs, serum, and brain regions across three life stages. Key findings include elevated I3P and Kyn levels in aging male livers and clear divergence in metabolite abundance across tissues and sexes. Trp restriction through diet significantly reduced circulating metabolites but had variable effects across organs, highlighting differential tissue buffering. This resource lays essential groundwork for understanding physiological Trp metabolism and its reprogramming in disease states, particularly in cancer and neurological disorders.

## Discussion

Despite extensive knowledge of RNA and protein expression across organs, tissues, and developmental stages, quantifying metabolites requires customized methods, ultimately limiting the use of high‐throughput analyses. Yet, studying metabolites has the potential to fundamentally shift our approach to understanding, diagnosing, and treating disease. Gaining a deeper understanding of the production and utilization of Trp metabolites in healthy tissues can lead to a better understanding of the deregulation of this pathway in pathological conditions. In the current study, we identified age‐, sex‐, and tissue‐specific variations in Trp metabolites, including the oncometabolites Kyn and I3P, which were highest in aging male mice. These findings suggest that dysregulation of Trp metabolism may not only result from disease but also contribute to disease susceptibility. Interestingly, while I3P levels were equally high in animals fed a control or Trp‐free diet the normal liver; our previous studies demonstrated that the Trp‐free diet led to a marked reduction of I3P in MYC‐driven liver tumors [19]. We surmise that this difference reflects a the different biology of normal liver and liver tumors [19]. Moreover, elevated Kyn levels in the colon with aging may correlate with increased risks of colorectal cancer and inflammatory bowel diseases, which are conditions with higher prevalence in aging males. Such inflammatory diseases and cancer are often associated with shifts in the gut microbiome. Interestingly, also numerous associations between Trp metabolite levels and neurological conditions have been identified. For example, low serotonin levels are linked to depression, and disruptions in metabolites like Kyn, XA, CA, and KA have been associated with various neurological disorders [5, 12, 22, 24, 25, 27, 28, 45, 47, 48]. Clarifying these connections through further studies may significantly improve the understanding of mental health disorders.

Although our study provides valuable insights into steady‐state Trp metabolite levels across tissues, ages, and sexes, significant gaps persist in understanding the mechanisms regulating the transport of Trp and its metabolites into specific cells and tissues. Metabolite presence in certain tissues may result from local enzymatic activity, transporter expression, or a combination of its uptake, synthesis, and stability. Elevated metabolite levels in an organ likely indicate their functional importance within that tissue. Therefore, identifying specific Trp metabolite transporters and targeting Trp‐metabolizing enzymes in precisely in particular organs may help to address these knowledge gaps, potentially facilitating the development of strategies to diagnose and treat disease associated with deregulated Trp metabolism. Additionally, dietary intake data may provide insights into how Trp (or its metabolites) availability from dietary sources impacts metabolite availability/production, potentially revealing dietary factors in disease prevention or risk.

## Limitations of the study

While our platform can accurately quantify 17 Trp metabolites, only 13 yielded distinguishable peaks across all tissue types and biological replicates. Figures display all metabolites measurable in replicates. While our Trp metabolite platform focuses on quantifying metabolites known to be produced in mammalian tissues, some of these metabolites may be generated by the microbiome. However, our analyses do not differentiate between host‐derived and microbiome‐derived metabolites, as several indoles and other microbiome‐specific compounds were not captured. Addressing microbiome contributions would require additional experiments involving microbiome depletion to assess their distinct impact, as well as the development of new methods specifically aimed at characterizing these metabolites. We plan expand our platform to address the deficiencies in the future. Finally, because of the large volume of tissue samples processed, not all extractions could be performed at the same time of day. A subset of samples was collected in the morning, while others were processed in the afternoon, which may introduce some temporal variability.

## Conflict of interest

The authors declare no conflict of intertest.

## Author contributions

MCS, LPC, RG planned the experiments. MCS, LPC, AFN, MCLN, RG wrote the manuscript. RG, MCLN, AFN, helped with manuscript edits. LPC, RG, PN, JK, AFN performed experiments. LPC, PA, JK, RG performed data analyses. NSW and MCS supervised.

## Supporting information


**Fig. S1.** Control curves for Trp‐derived metabolite abundance.

**Fig. S2.** Abundance of Trp metabolites across different organs in 3‐week‐old mice tissues compared to serum.

**Fig. S3.** Abundance of Trp metabolites across different organs in 74‐week‐old mice tissues compared to serum.

**Fig. S4.** Trp metabolites differences by age.

**Fig. S5.** Trp metabolite level changes in aging stratified per metabolite.

**Fig. S6.** Trp metabolites differences in the brain.

**Table S1.** Quantification of Trp metabolite contents in the defined amino acid diet (AA) and Trp‐free diet (TF), measured by LC–MS/MS.

**Table S2.** Resources used in this study.

Data S1.

## Data Availability

Further information and requests for resources and reagents (Table S2) should be directed to and will be fulfilled by the Lead Contact: Maralice Conacci‐Sorrell (maralice.conaccisorrell@utsouthwestern.edu). This study did not generate new unique reagents and does not report original code. All information necessary to reanalyze the data reported in this paper is provided in the two accompanying raw data files attached.
