# Supplementary material for: Tryptophan metabolite atlas uncovers organ, age, and sex‐specific variations
Source: FEBS Open Bio. 2025 Sep 19;16(1):52–67. doi: 10.1002/2211-5463.70123 (PMC12767773; doi:10.1002/2211-5463.70123)
Supplement: Supplementary file 1 — Fig. S1. Control curves for Trp‐derived metabolite abundance. [file FEB4-16-52-s006.pdf]

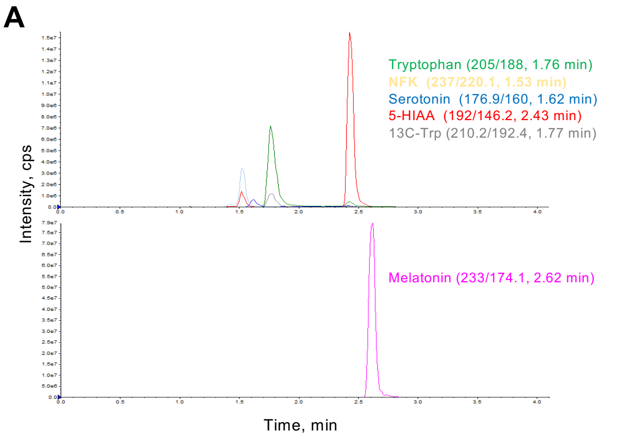

Tryptophan, NFK (N-formyl kynurenine), Serotonin, 5-HIAA (5-hydroxyindoleacetic acid), 13C-Tryptophan, and Melatonin, 1000 ng/mL each, run together in a standard curve.

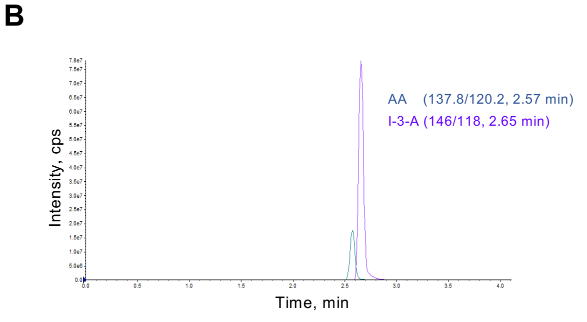

AA (Anthranilic acid) and I-3-C (Indole-3-carboxyaldehyde), 100 ng/mL each, run together in a standard curve

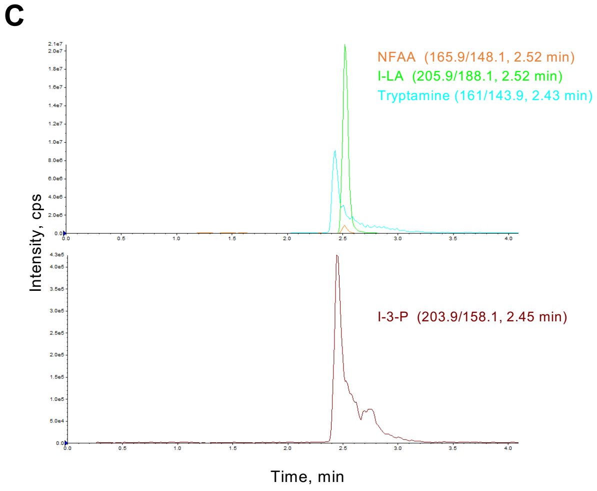

NFAA (N-formylanthranilic acid), I-LA (Indole-3-lactic acid), and Tryptamine, 100 ng/mL each; I-3-P (Indole-3-pyruvic acid), 1000 ng/mL, run together in a standard curve

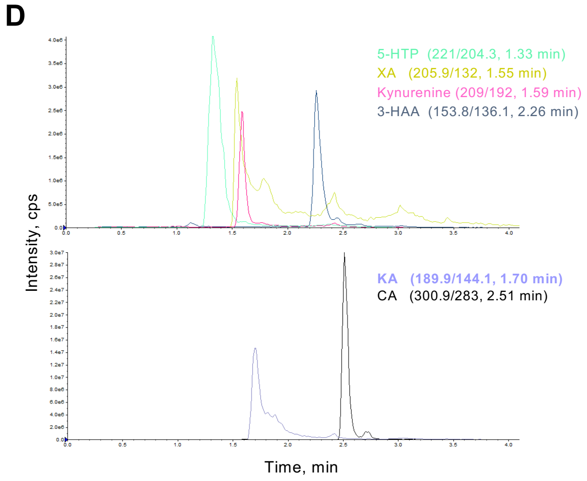

Kynurenine, 3-HAA (3-hydroxyanthranilic acid), XA (Xanthurenic acid), and 5-HTP (5-hydroxytryptophan), 5000ng/mL each; KA (Kynurenic acid) and CA (Cinnabarinic acid), 1000 ng/mL each, run together in a standard curve

Figure S1: Control curves for Trp-derived metabolite abundance.

(A-D) Method developed to measure the indicated Trp metabolites by high-performance liquid chromatography (HPLC)-tandem mass spectrometry (LC-MS/MS); the indicated Trp metabolites (1 ng/mL) were spiked into tissue lysates, and the elution time for each was obtained.

(A) Running time by LC-MS/MS of the metabolites Trp, NFK, Serotonin, 5HIAA and Melatonin.

(B) Running times by LC-MS/MS of AA and I3A.

(C) Running times by LC-MS/MS of NFAA, ILA, Tryptamine and I3P.

(D) Running time by LC-MS/MS of the metabolites 5HTP, Xa, Kyn, 3HAA, KA, and CA.
