# Supplementary material for: Tryptophan metabolite atlas uncovers organ, age, and sex‐specific variations
Source: FEBS Open Bio. 2025 Sep 19;16(1):52–67. doi: 10.1002/2211-5463.70123 (PMC12767773; doi:10.1002/2211-5463.70123)
Supplement: Supplementary file 3 — Fig. S3. Abundance of Trp metabolites across different organs in 74‐week‐old mice tissues compared to serum. [file FEB4-16-52-s007.pdf]

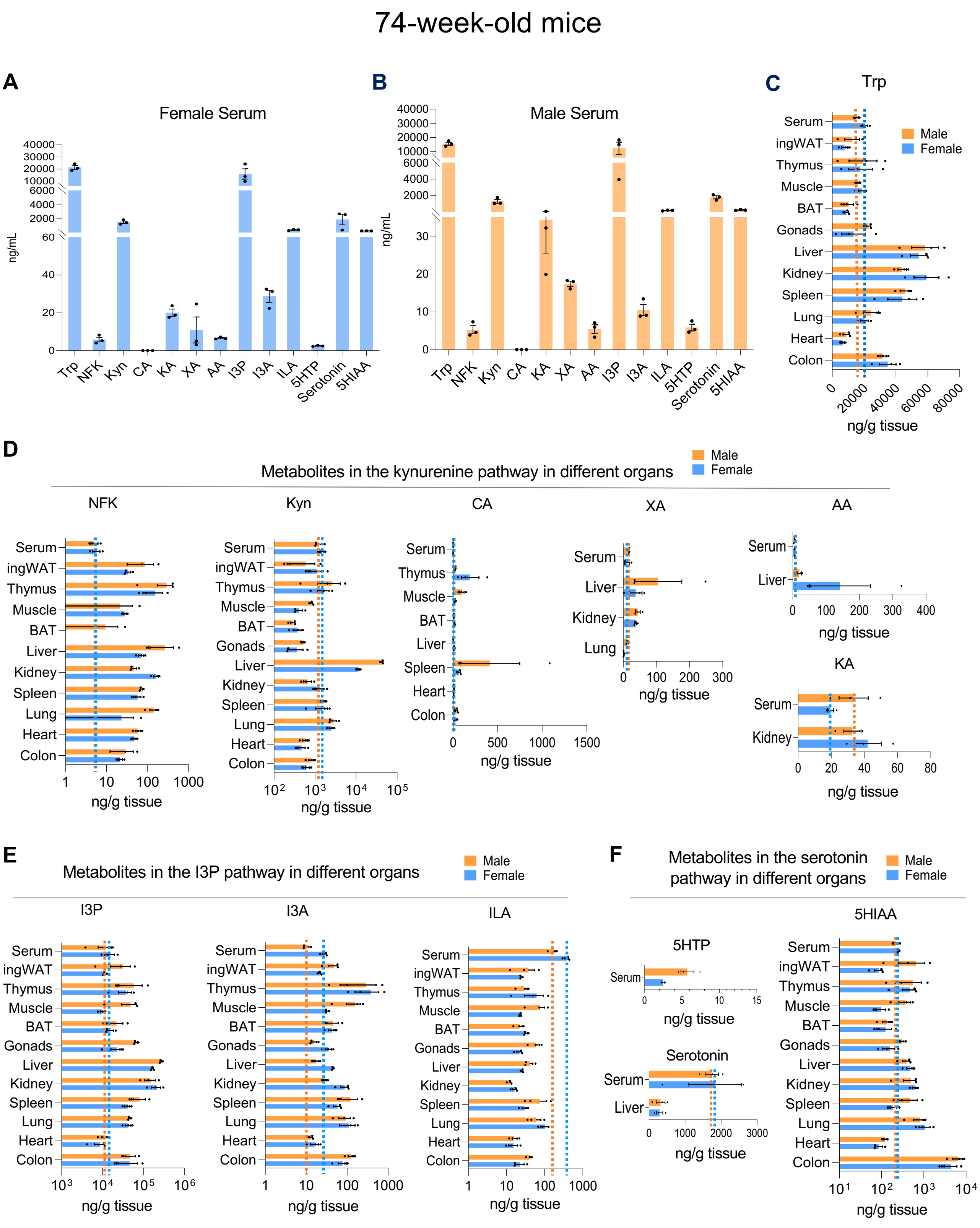

Figure S3: Abundance of Trp metabolites across different organs in 74-week-old mice tissues compared to serum.

(A) Abundance (ng/mL) of all Trp-derived metabolites by LC-MS/MS across different tissues in the serum of females.

(B) Abundance (ng/mL) of all Trp-derived metabolites by LC-MS/MS across different tissues in the serum of males.

(C) Abundance (ng/g of tissue) of Trp metabolite across all tissues compared to the amount circulating in serum (ng/g) (dotted line).

(D) Abundance (ng/g of tissue) of Kyn pathway metabolites across all tissues compared to the amount circulating in serum (ng/g) (dotted line).

(E) Abundance (ng/g of tissue) of I3P pathway metabolites across all tissues compared to the amount circulating in serum (ng/g) (dotted line).

(F) Abundance (ng/g of tissue) of serotonin pathway metabolites across all tissues compared to the amount circulating in serum (ng/g) (dotted line). The density of mouse serum was estimated to be approximately 1.025 g/mL, which is consistent with reported values for mammalian serum. Using this density, all measurements were converted from ng/mL (per volume) to ng/g (per mass) to allow for more accurate comparisons across samples.

(A-F) Errors bars indicate the mean with SEM; Females N=3, Males N=3
