# Supplementary material for: Tryptophan metabolite atlas uncovers organ, age, and sex‐specific variations
Source: FEBS Open Bio. 2025 Sep 19;16(1):52–67. doi: 10.1002/2211-5463.70123 (PMC12767773; doi:10.1002/2211-5463.70123)
Supplement: Supplementary file 4 — Fig. S4. Trp metabolites differences by age. [file FEB4-16-52-s001.pdf]

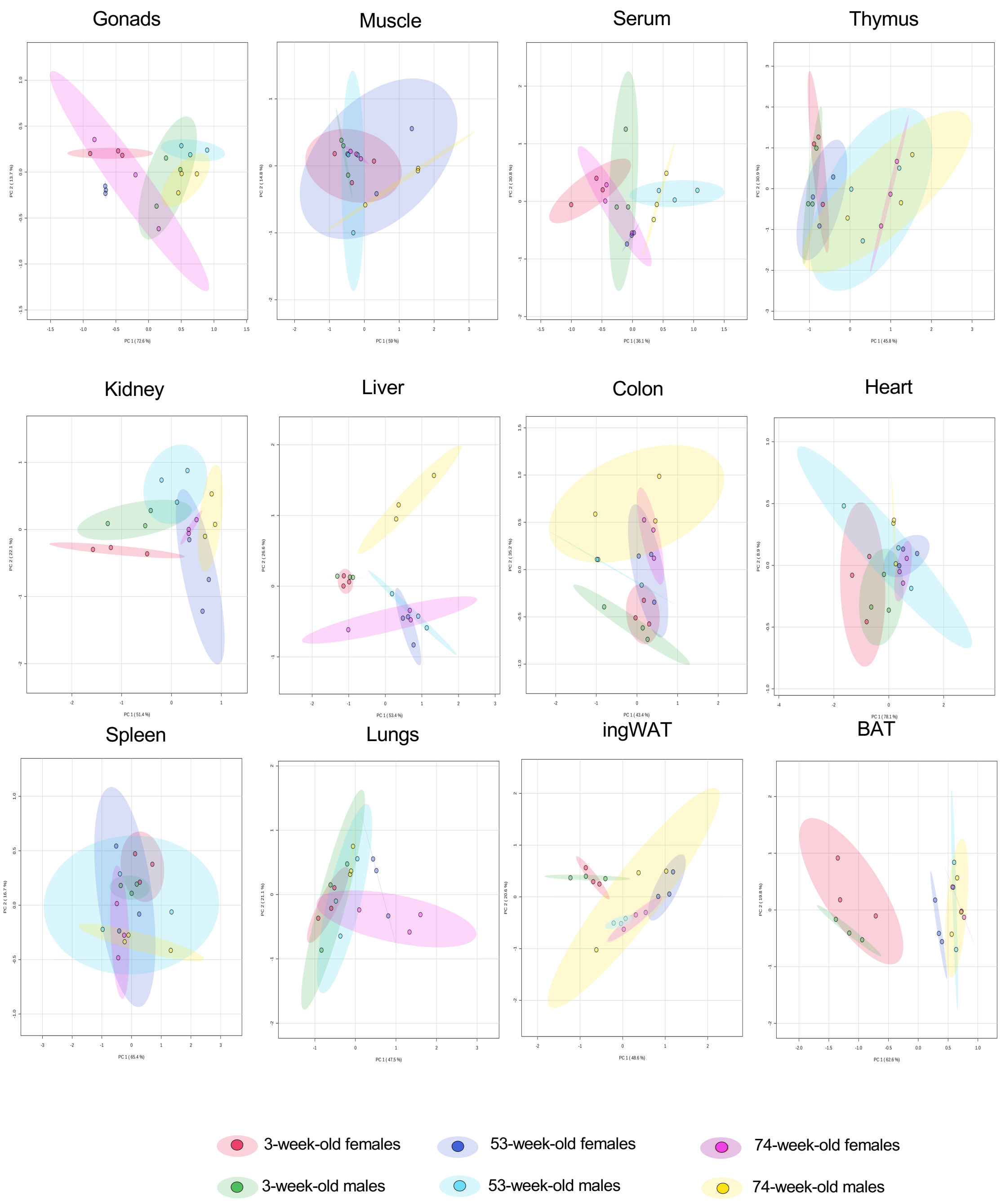

Figure S4: Trp metabolites differences by age.

2D PCA plots of all metabolites per organ and age. Some organs like Liver and BAT show different clustering of the metabolites by age. This suggests possible aging effects with certain Trp-derived metabolites.
