# Supplementary material for: Tryptophan metabolite atlas uncovers organ, age, and sex‐specific variations
Source: FEBS Open Bio. 2025 Sep 19;16(1):52–67. doi: 10.1002/2211-5463.70123 (PMC12767773; doi:10.1002/2211-5463.70123)
Supplement: Supplementary file 5 — Fig. S5. Trp metabolite level changes in aging stratified per metabolite. [file FEB4-16-52-s009.pdf]

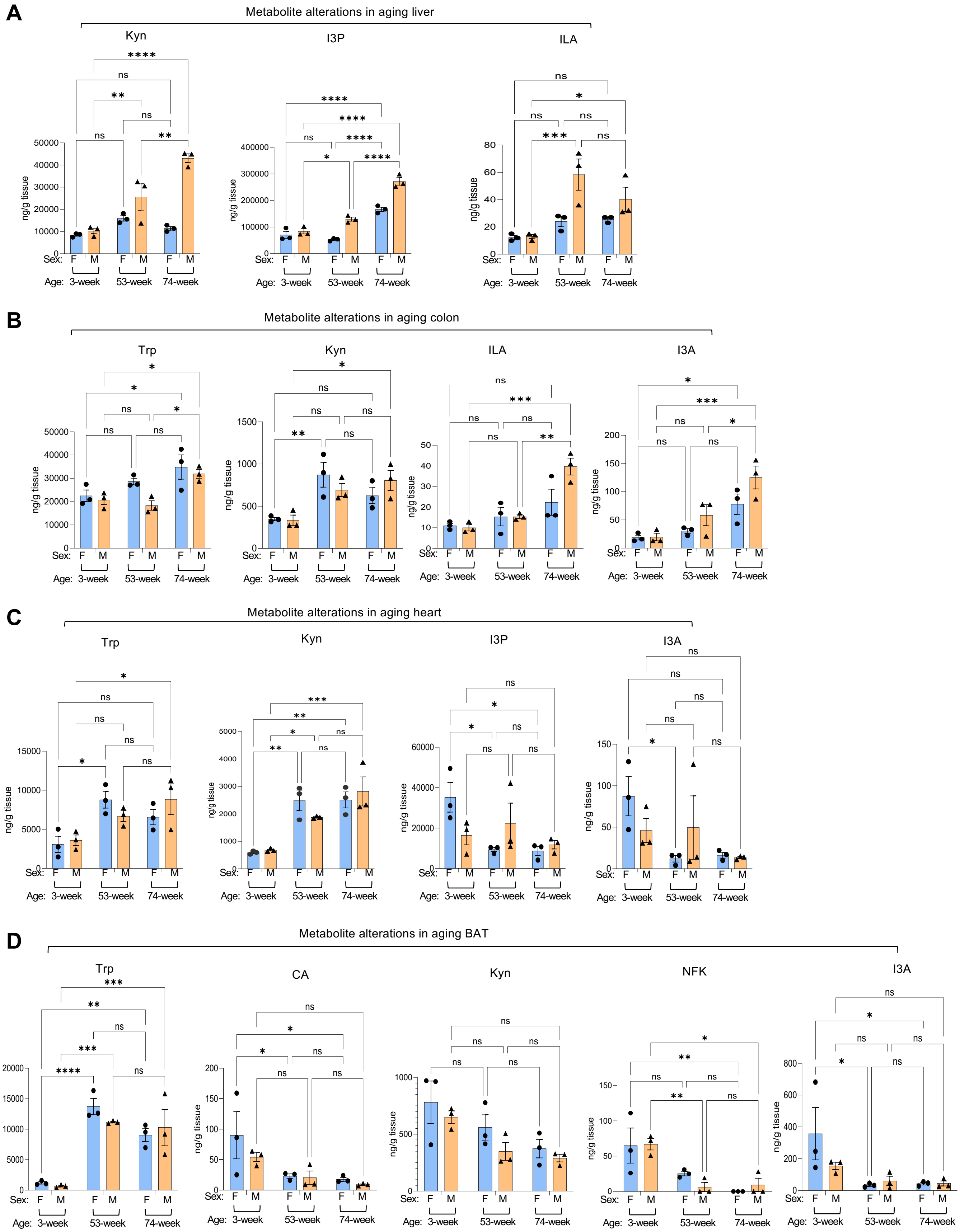
A
Metabolite alterations in aging liver
Kyn
I3P
ILA
ng/g tissue
Sex: F M F M F M
Age: 3-week 53-week 74-week
B
Metabolite alterations in aging colon
Trp
Kyn
ILA
I3A
ng/g tissue
Sex: F M F M F M
Age: 3-week 53-week 74-week
C
Metabolite alterations in aging heart
Trp
Kyn
I3P
I3A
ng/g tissue
Sex: F M F M F M
Age: 3-week 53-week 74-week
D
Metabolite alterations in aging BAT
Trp
CA
Kyn
NFK
I3A
ng/g tissue
Sex: F M F M F M
Age: 3-week 53-week 74-week

Figure S5: Trp metabolite level changes in aging stratified per metabolite.

(A) Significant changes in abundance for Trp (Kyn, I3P, ILA) metabolites across aging in the male liver. (B) Significant changes in abundance of Trp (Trp, Kyn, I3P, ILA, I3A) metabolites across aging in the colon.

(C) Significant changes in abundance of Trp (Trp, Kyn, I3P, I3A) metabolites across aging in the heart.

(D) Significant changes in abundance of Trp (Trp, Kyn, CA, NFK, I3A) metabolites across aging in the BAT.

(A-D) Statistical analysis was performed using 2-way ANOVA (Tukey's multiple tests comparison) on all age groups.
