# Supplementary material for: Tryptophan metabolite atlas uncovers organ, age, and sex‐specific variations
Source: FEBS Open Bio. 2025 Sep 19;16(1):52–67. doi: 10.1002/2211-5463.70123 (PMC12767773; doi:10.1002/2211-5463.70123)
Supplement: Supplementary file 6 — Fig. S6. Trp metabolites differences in the brain. [file FEB4-16-52-s005.pdf]

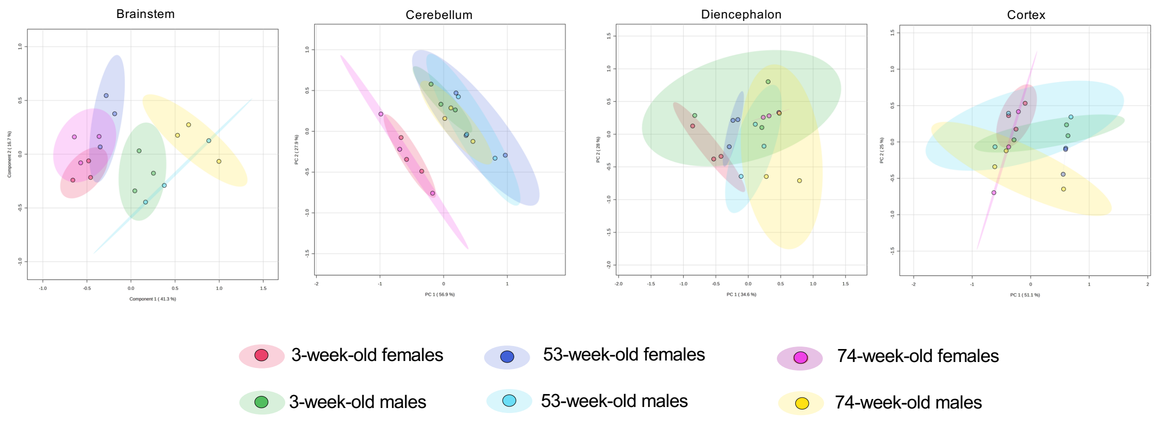

Figure S6: Trp metabolites differences in the brain.

2D PCA plots showing all metabolites per brain region. The brain regions did not show any aging effects, but a clustering in the cerebellum by sex shows some differences in the abundance of Trp-derived metabolites.
