# Supplementary material for: Tryptophan metabolite atlas uncovers organ, age, and sex‐specific variations
Source: FEBS Open Bio. 2025 Sep 19;16(1):52–67. doi: 10.1002/2211-5463.70123 (PMC12767773; doi:10.1002/2211-5463.70123)
Supplement: Supplementary file 7 — Table S1. Quantification of Trp metabolite contents in the defined amino acid diet (AA) and Trp‐free diet (TF), measured by LC–MS/MS. [file FEB4-16-52-s008.pdf]

Table S1. Quantification of Trp metabolite contents in the defined amino acid diet (AA) and Trp-free diet (TF), measured by LC-MS/MS

**Metabolites**	**Diets**	
	**AA** (ng/g)	**TF** (ng/g)
Trp	1474940.5	393.3
NFK	552.8	11.0
Kyn	5260.8	66.0
KA	3627.3	8.3
AA	19.3	2.8
NFAA	0	0
XA	188.9	191.1
3HAA	0	0
CA	649.0	30.3
I3P	3250.5	2475.0
ILA	324.5	33.0
I3A	522.5	11.0
5HTP	214.5	2.8
Serotonin	1534.5	409.8
5HIAA	211.8	115.5
Melatonin	0	0
Tryptamine	1193.5	88.0
