# Supplementary material for: Tryptophan metabolite atlas uncovers organ, age, and sex‐specific variations
Source: FEBS Open Bio. 2025 Sep 19;16(1):52–67. doi: 10.1002/2211-5463.70123 (PMC12767773; doi:10.1002/2211-5463.70123)
Supplement: Supplementary file 8 — Table S2. Resources used in this study. [file FEB4-16-52-s003.pdf]

Table S2: Resources used in this study

REAGENT or RESOURCE	SOURCE	IDENTIFIER
Chemicals		
Tryptophan ($C_{11}H_{12}N_2O_2$)	Sigma	T8941-25G
N-formylkynurenine ($C_{11}H_{12}N_2O_4$)	Santa cruz biotechnology	sc-490237
Kynurenine ($C_{10}H_{12}N_2O_3$)	Sigma	K8625-25MG
Indole-3-pyruvic acid ($C_{11}H_9NO_3$)	Sigma	I7017-1G
Indole-3- carboxyaldehyde (C_9H_7NO)	Sigma	129445-5G
Indole-3-lactic acid ($C_{11}H_{11}NO_3$)	Sigma	I5508-250MG-A
5-Hydroxyindole-3-acetic acid ($C_{10}H_9NO_3$)	Sigma	55697-50MG
Tryptamine ($C_{10}H_{12}N_2$)	Sigma	193747-10G
5-Hydroxytryptophan ($C_{11}H_{12}N_2O_3$)	Sigma	399698-25GM
N-formyl anthranillic acid ($C_8H_7NO_3$)	Sigma	S836886-250MG
Anthranillic acid ($C_7H_7NO_2$)	Sigma	A89855-25G
3-Hydroxyanthranilic acid ($C_7H_7NO_3$)	Cayman Chemicals	20512
Cinnabarinic acid ($C_{14}H_8N_2O_6$)	Cayman Chemicals	11988
Melatonin ($C_{13}H_{16}N_2O_2$)	Cayman Chemicals	14427
Serotonin ($C_{10}H_{12}N_2O$)	Sigma	14927
Xanthurenic acid ($C_{10}H_7NO_4$)	Cayman Chemicals	19191
L-Tryptophan-2',4',5',6',7'-d5 (C11H7D5N2O2)	CDN Isotopes	D-1522
Indole-3-acetic Acid-d5 (C10H4D5NO2)	Cayman Chemicals	38894
Experimental models: Organisms/strains		
C57BL/6 mice	Jackson lab	#000664
Software and algorithms		
Prism 9	Graphpad	N/A
Spyder		
Metaboanalyst		
Analyst 1.7.2	AB Sciex	
Other		
Nexera X2<br>HPLC System	Shimadzu	
QTrap 6500+ Mass Spec	Sciex	
Synergi Polar-RP 2.0 X 100mm, 2.5 micron packing	Phenomenex	00D-4371-B0
